# 

**Published:** 1919-11-01

**Authors:** 


### THE HOSPITAL

### The Modern Newspaper of Administrative Medicine and Institutional Life.

Telegraphic Address: OSPEDALE, RAND, LONDON. Telephone Number: 2734 GERRARD

No. 1743, Vol. LXVII. | Saturday, November 1st, 1919. | PRICE TWOPENCE

